# Mammalian Oocyte Analysis by MALDI MSI with Wet-Interface Matrix Deposition Technique

**DOI:** 10.3390/ma16041479

**Published:** 2023-02-09

**Authors:** Anna Bodzon-Kulakowska, Wiesława Młodawska, Przemyslaw Mielczarek, Dorota Lachowicz, Piotr Suder, Marek Smoluch

**Affiliations:** 1 Department of Analytical Chemistry and Biochemistry, Faculty of Materials Science and Ceramics, AGH University of Science and Technology, A. Mickiewicza 30, 30-059 Krakow, Poland; 2 Department of Animal Reproduction, Anatomy and Genomics, University of Agriculture in Krakow, Al. Mickiewicza 24/28, 30-059 Krakow, Poland; 3 Laboratory of Proteomics and Mass Spectrometry, Maj Institute of Pharmacology, Polish Academy of Sciences, Smetna 12, 31-343 Krakow, Poland; 4 Academic Centre for Materials and Nanotechnology, AGH University of Science and Technology, A. Mickiewicza 30, 30-059 Krakow, Poland

**Keywords:** oocytes, MALDI, imaging, single cell, lipidomics, wet-interface

## Abstract

Oocytes are a special kind of biological material. Here, the individual variability of a single cell is important. It means that the opportunity to obtain information about the lipid content from the analysis of a single cell is significant. In our study, we present a method for lipid analysis based on the MALDI-based mass spectrometry imaging (MSI) approach. Our attention was paid to the sample preparation optimization with the aid of a wet-interface matrix deposition system (matrix spraying). Technical considerations of the sample preparation process, such as the number of matrix layers and the position of the spraying nozzle during the matrix deposition, are presented in the article. Additionally, we checked if changing the 2,5-dihydroxybenzoic acid (DHB) and 9-Aminoacridine (9AA) matrix concentration and their solvent composition may improve the analysis. Moreover, the comparison of paraformaldehyde-fixed versus nonfixed cell analysis was performed. We hope that our approach will be helpful for those working on lipid analyses in extraordinary material such as a single oocyte. Our study may also offer clues for anybody interested in single-cell analysis with the aid of MALDI mass spectrometry imaging and the wet-interface matrix deposition method.

## 1. Introduction

The process of oocyte maturation and the acquisition of developmental competence is complex, regulated by many factors linked to oocyte metabolism, and is still not fully understood. Research suggests that not only carbohydrates but also lipids are used as fundamental energy donors for oocytes during development and maturation [1,2,3]. In general, in mammalian oocytes lipids are mainly stored in the form of lipid droplets (LDs) containing mostly phospholipids (PLs), neutral triacylglycerols (TAGs), and sterol esters [4,5,6,7]. Lipid content and composition undergo dynamic changes during folliculogenesis and oocyte final maturation [1,8]. Total lipid composition, type of lipids, and fatty acid (FAs) content of oocytes have been shown to differ significantly among mammalian species [9,10]. It is generally accepted that lipid-rich oocytes of pigs and cats have visually darker appearance in bright-field microscopy than those with smaller intracellular lipid stores, for example, in mice and rats [6,10,11]. Even oocytes derived from the same ovary may visually differ with respect to ooplasm appearance/darkness level. Studies have shown that bovine oocytes classified into different “darkness categories” differ in the proportion of certain unsaturated (oleic, linoleic, and arachidonic) and saturated (stearic) FAs content [12] and after in vitro fertilization (IVF) displayed different capacity for cleavage and blastocyst formation in vitro [13].

Unfortunately, “darkness level” is an extremely inaccurate type of measurement, even if somehow connected to the lipid composition in the cells. Therefore, getting to know the composition of intracytoplasmic lipids is essential for a deeper understanding of the role of lipids and their metabolism in controlling the mechanisms of oocyte development. Such knowledge creates the possibility to improve oocyte quality by modulating lipid metabolism (for example, by the addition of specific lipid compounds to culture media) and may find application in increasing the efficiency of in vitro embryo production in mammals.

For lipid study and visualization in mammalian oocytes various techniques are used, each having its advantages and limitations, enabling lipid analysis in different aspects. Exemplary, gas chromatography allows for the evaluation of the total lipid content, the ratio of FAs content, and composition. However, this technique is expensive and, for successful analysis, requires a large number (usually several hundred) of oocytes. Fluorescent staining, using such dyes as Nile red or BODIPY 493/503 in association with confocal laser microscopy analysis, is commonly used for LDs imaging and semi-quantification in a single oocyte. However, this approach failed to provide detailed information about the different types and content of accumulated lipids. Electron microscopy allows the visualization and assessment of the position, size, and form of LDs within a single cell but does not provide any information about lipid species or their content (for review, see [14]).

Proposed here, MALDI (matrix-assisted laser desorption ionization) mass spectrometry imaging originates from the mass spectrometry-based analytical techniques, allowing for the simultaneous analysis of the majority of lipids building the cell membrane of a single oocyte, as well as the lipid composition of the cytoplasm. MALDI-MSI is a very sensitive method, detecting molecules at the pico- or even femtomolar level, which makes it the method of choice for lipid analysis from a single cell (for concise review, see: [15]). Furthermore, as it is possible to measure, almost simultaneously, various cells placed on the glass target plate one after another, it is possible to easily compare a constituent of different kinds of oocytes. Finally, it allows us to understand the biological background of the differences detected between them.

The MALDI-based analysis uses the so-called matrix, being the weak organic acid (like dihydroxybenzoic, cinnamic, or others), which must be applied onto the sample to be imaged. Matrix spraying and matrix sublimation are currently the two most popular matrix deposition techniques. During matrix sublimation, no liquid phase is used, which should minimize the problem of molecules delocalization in the tissue. For lipids, it usually offers a significant enhancement of the signal acquired [16]. Due to the characteristics of sublimation methods (the ease of preparation, the purification of matrix during sublimation, and no need for expensive devices), they are still under development. This means that the new devices that are invented offer better control of the parameters of matrix deposition [17,18]

Spraying techniques offer more efficient analyte extraction and crystallization, essential when protein analysis is considered. Moreover, the possibility of manipulating spraying parameters offers the option of matrix deposition similar to the sublimation process. Devices for matrix spraying are expensive but, in the meantime, they are more versatile.

The efficient methodology of oocyte measurements using MALDI-MSI was proposed and used by our team previously [7,19]. This approach was based on the older system for matrix deposition, ImagePrep^TM^, which uses ultrasonic spraying from the metallic surface.

The aim of the study presented here is to optimize the lipid analysis from the single oocyte with the aid of SunCollect system. It is recognized as the “wet-interface matrix deposition method” and is based on an optimized and highly efficient spray generator that produces extremely small matrix droplets. The efficient coverage of the cells by the matrix prior to MALDI-MSI analysis is one of the most important optimization steps, so, in our opinion, it deserves to be described in detail. Our remarks could be useful for those who would like to use MSI technology in cellular lipids’ qualitative and semi-quantitative measurements.

During covering the oocytes with the SunCollect set-up, the number of matrix layers and the distance from the spraying tip to the cells could be adjusted. Z = 1 mm is the highest position above the cells, and Z = 49 mm is the lowest position possible. Spraying the matrix from the topmost position results in a very dry spray, while the lowest position results in a wetter vapor and better solution penetration into the sample. It should also be remembered that the cells must be deposited onto a special surface before the matrix deposition: indium-tin-oxide covered glass (ITO glass). A conductive surface just under the cells is necessary for the ionization of the molecules during the measurement, while a transparent plate makes it easier to find the cells on the surface during the ionization process.

To optimize MS-based imaging results, we tested two MALDI matrices, selected by us previously as perfect for investigations on oocytes: 2,5-dihydroxybenzoic acid (DHB) for positive ionization mode and 9-aminoacridine (9AA) for negative ionization mode. Different ionization modes make a wider identification range of various kinds of lipids. In the positive ionization mode, mainly glycerophosphocholines and sphingomyelins are detected. Meanwhile, the negative ionization mode is suitable mainly for glycerophosphoethanolamines, glycerophosphoserines, and glycerophosphoinositols. The other lipids can also be detected depending on the sample properties.

In the case of both modes, four different positions of the spraying nozzle above the sample surface and the influence of the number of the matrix layers on the quality of obtained MS spectra were examined. Additionally, we have tested how the matrix concentration and the type of matrix solvent influence the results. Moreover, oocytes briefly washed with water were compared in those fixed with 4% paraformaldehyde (PFA) using MALDI-MSI and AFM (Atomic Force Microscope).

## 2. Materials and Methods

### 2.1. Oocyte Collection and Preparation

The oocytes were collected from female domestic cat (*Felis catus*) ovaries after a routine sterilization procedure performed at private local veterinary surgery and animal shelter (Krakow, Poland). The ovaries were transported to the laboratory in phosphate-buffered saline (PBS; Biomed, Lublin, Poland) supplemented with penicillin (100 IU/mL) and streptomycin (100 µg/mL) at 4 °C [20]. Cumulus-oocyte complexes (COCs) were recovered by slicing the ovaries in OPU-medium (Bioscience, Bickland Industrial Park, Falmouth, Cornwall, UK), washed in PBS and denuded of surrounding cumulus cells by mechanical pipetting, and kept in PBS at 4 °C up to 12 h. The oocytes were then: (i) washed three times with deionized water to remove the salts and buffer compounds (PBS-group) or (ii) fixed by incubating in 4% paraformaldehyde (PFA-group) dissolved in PBS for 15 min, followed by washing thrice in ultrapure water. Subsequently, the oocytes were deposited on the ITO glass (Bruker-Daltonics, Bremen, Germany), near the white mark from the nail polish, facilitating the cell localization on the glass slide during MALDI analysis. The glass surface was covered with the appropriate MALDI matrix, which allowed for the desorption and ionization of the molecules present in the analyzed sample. To be able to properly localize the oocytes on the ITO glass, a picture of each oocyte under the binocular magnifier or the microscope was taken and included in some of the Figures.

### 2.2. MALDI Matrices

During the experiments, the following chemicals were used. Solvents: acetonitrile (ACN) and methanol (J.T. Baker, Amsterdam, The Netherlands), both at the HPLC gradient grade, ethanol (Avantor, Poland), ultrapure water (Sigma-Aldrich/Merck, Darmstadt, Germany). Matrices: 2,5-dihydroxybenzoic acid (DHB), trifluoroacetic acid (TFA), 9-aminoacridine (9AA), all from Sigma-Aldrich/Merck.

### 2.3. Matrix Application

Before the MALDI analysis, an optical image of a glass slide with oocytes and marked fiducials—to be able to localize the sample—was taken. Then, the matrices were applied by a SunCollect device (SunChrom GmbH, Friedrichsdorf, Germany). For each matrix, a different number of layers were applied. The Z value—the position of the spraying nozzle above the tissue surface was also tested for different matrices. Z = 1 means the highest possible (about 50 mm) position above the tissue, whereas Z = 25 means the position about 25 mm above the tissue and is determined as the “extraction mode”.

The flow rate of the matrix solution was variable among layers, according to the manufacturer recommendations: 10 µL/min for the first layer, 20 µL/min for the second layer, 30 µL/min for the third layer, 40 µL/min for the fourth layer, and 60 µL/min for all other layers. The nozzle applied the matrix solution with a line distance of 2 mm, and speed equals 600 mm/min. At the beginning, for each matrix, four different numbers of layers and four different nozzle heights were tested. Then, the best parameters were used for different solvents of the same matrix in different concentrations.

### 2.4. MALDI Measurements

Matrix-coated area containing deposited oocytes were subjected to imaging experiments using the MALDI–TOF/TOF UltrafleXtreme MS (Bruker-Daltonics, Bremen, Germany) with a Smartbeam II™ laser (Bruker-Daltonics) operating at 2 kHz. All following MS parameters underwent initial, multistep optimization. Ions were accelerated at 25 kV with a pulsed ion extraction of 120 ns and ion suppression up to 100 Da. Spectra were recorded in positive and negative ionization modes with reflectron within a 200–3000 *m*/*z*. They were externally calibrated with Peptide Calibration Standard II (Bruker Daltonics, Bremen, Germany) and known matrix ions, e.g., 273.03990 *m*/*z* for DHB matrix ([2DHB-2H_2_O+H]^+^ ion) and 229.05330 *m*/*z* for 9AA matrix ([9AA+Cl]^−^ ion). A raster width of 50 μm was applied to all samples. In total, 400 shots were collected from each ablation point with 20 shots at the raster spot, and the laser focus diameter was set to “3_medium”. FlexControl version 3.4 (Bruker Daltonics) was employed for spectra acquisition, and FlexImaging version 4.0 was used for data processing and the creation of molecular images. Mmass software (version 5.5.0, Open-Source software developed by Martin Strohalm, Academy of Sciences, Prague, Czech Republic) was used for the spectra analysis [21].

### 2.5. Assessment of the Results

**Figure 1:**
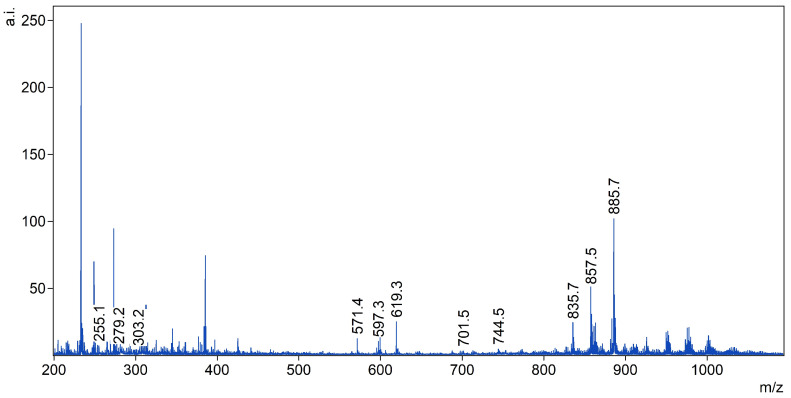
Peaks chosen for the analysis in the negative ionization mode (marked by their *m*/*z* ratios).

For the purpose of our analysis, peaks that represent the main groups of molecules that could be detected in the different ion modes and are widely recognized in the literature were chosen. For the negative ionization mode single fatty acids (255 (16:0), 279 (18:2), 303 (20:4)), lysophosphatidylinositols (LPI) (571 (LPI 16:0), 597 (LPI 18:1), 619 (LPI 20:4), phosphatidic acid 701.5 (PA 36:1), phosphatidylethanolamines (PE) (744.5 (PE 36:1), 762.5 (PE 38:6)) and phosphoinositides (PI) (835 (PI 34:1), 857 (PI 36:4), 885 (PI 38:4)) were selected (see Figure 1).

**Figure 2:**
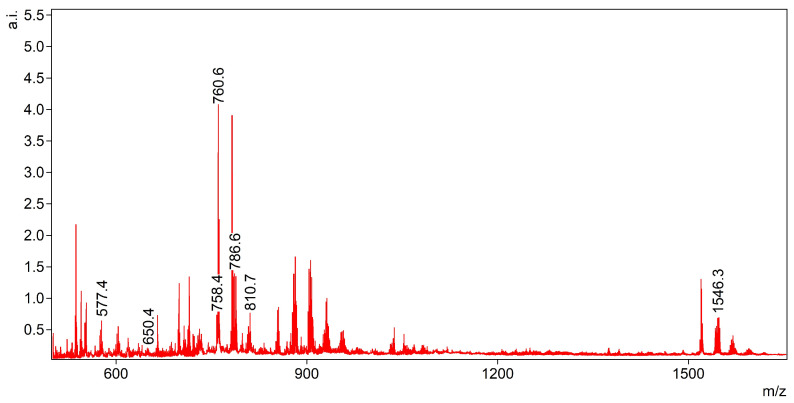
Peaks chosen for the analysis in the positive ionization mode (marked by their *m*/*z* ratios).

For the positive ionization mode, lysophosphatidylcholine 577.5 (LPC 22:1), and phosphatidylcholines (756.4 (PC 32:0), 758.6 (PC 34:2), 760.5 (PC 34:1), 782.5 (PC 36:4), 786.6 (PC 36:2), 808.6 (PC 36:2 Na), 810.7 (PC 36:1 Na), *m*/*z* 1546.8 were selected (see: Figure 2).

### 2.6. AFM Analysis

The morphology of the prepared oocyte samples was studied using atomic force microscopy (AFM). AFM topography images were obtained with Dimension Icon XR microscope (Bruker, Santa Barbara, CA, USA) working in the air in the PeakForce Tapping (PFT) mode using soft silicon cantilevers of nominal spring constant of 0.4 N/m and triangular geometry tip with a nominal tip radius of 2 nm. AFM data have been processed using NanoScope Analysis 1.9 and Gwyddion 2.6 software.

## 3. Results

### 3.1. Nozzle Height and the Number of Matrix Layers

#### 3.1.1. Positive Ionization Mode

**Figure 3:**
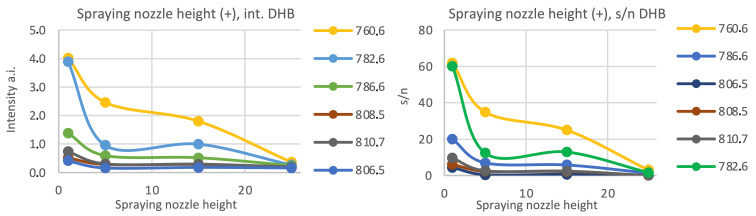
The relationship between the chosen peaks’ intensities and signal-to-noise ratio (s/n) in terms of the spraying nozzle height for the positive ionization mode, matrix: 25 mg/mL DHB (50% MeOH, 0.2% TFA), number of matrix layers: ten (L = 10).

**Figure 4:**
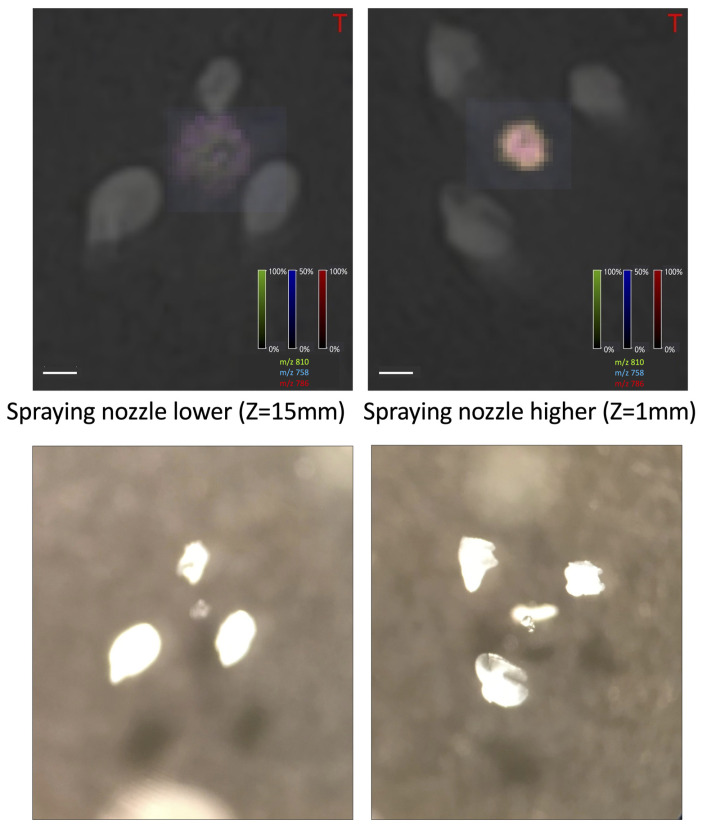
The picture of the oocyte obtained after matrix application at a lower nozzle position (Z = 15) and higher nozzle position (Z = 1), positive ionization mode, number of matrix layers: ten (L = 10), size bar: 200 μm). At lower position the content of the cell is going out of it (“splashing”). Below it is presented the microscopic image of oocytes.

In the positive ionization mode, four different positions of the spraying nozzle above the sample surface were tested. The intensity of the peaks increases with the height of the nozzle (see Figure 3). We also observed that lower nozzle height could be responsible for the unwanted “splashing” of the cell (see Figure 4).

**Figure 5:**
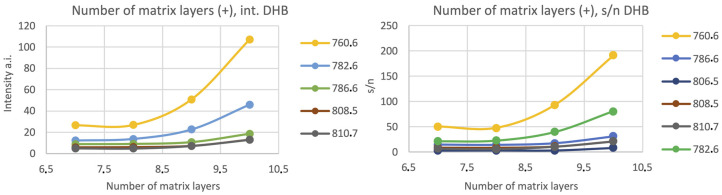
The relationship between the chosen peaks’ intensities and signal-to-noise ratio (s/n) in terms of the number of matrix layers for the positive ionization mode, matrix: 25 mg/mL DHB (50% MeOH, 0.2% TFA), nozzle position: Z = 25.

**Figure 6:**
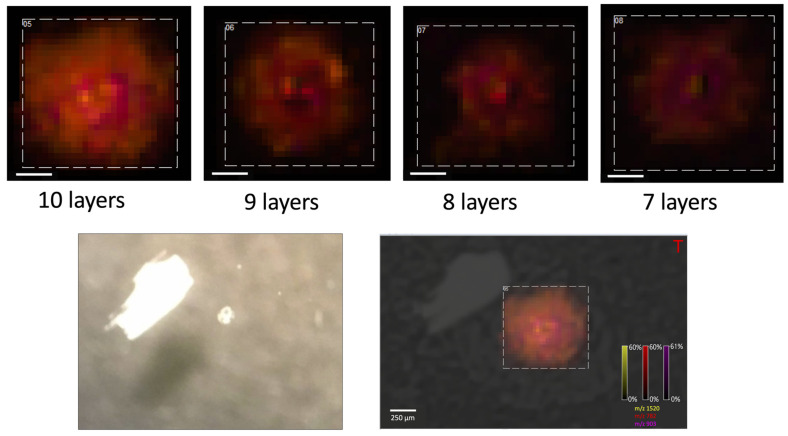
The MALDI-MSI picture of the oocyte obtained after covering oocytes with different numbers of the matrix layers, positive ionization mode, (*m*/*z* 810, nozzle position: Z = 25), size bar: 200 μm. Below it is presented the microscopic image of oocytes.

The influence of the number of the matrix layers on the quality of obtained MS spectra was also examined. The signal intensity increased with the number of layers for the peaks identified in the literature as derived from lipids (see: Figure 5 and Figure 6).

#### 3.1.2. Negative Ionization Mode

**Figure 7:**
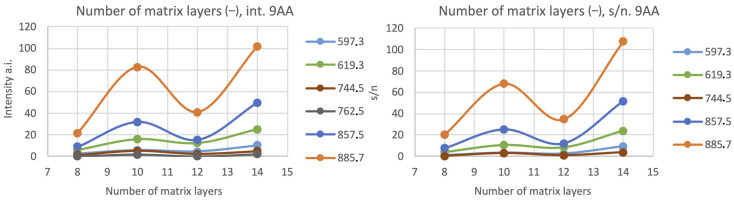
The relationship between the chosen peaks’ intensities and signal-to-noise ratio (s/n) in terms of the number of matrix layers for the negative ionization mode, matrix: 9AA 7 mg/mL 70% EtOH, nozzle position: Z = 25.

**Figure 8:**
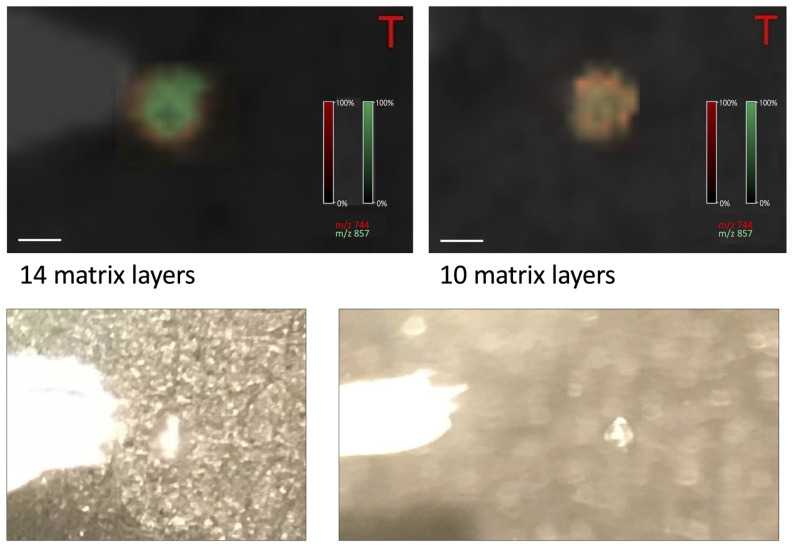
The picture of the oocytes obtained after covering them with different number of the matrix layers—negative ionization mode (*m*/*z* 744 and 857), size bar: 200 μm. Below it is presented the microscopic image of oocytes.

For the negative ionization mode, the number of applied matrix layers was also important, but not so obvious as for the positive ionization mode. For the oocytes washed with PBS, the most intense signal was with 14 matrix layers, and the signal-to-noise ratio was also quite high (see: Figure 7 and Figure 8).

**Figure 9:**
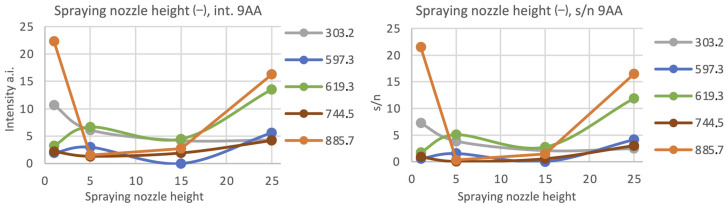
The relationship between the chosen peaks’ intensities and signal-to-noise ratio (s/n) in terms of the spraying nozzle height for the negative ionization mode, matrix: 9AA 7 mg/mL 70% EtOH, number of matrix layers: L = 14.

**Figure 10:**
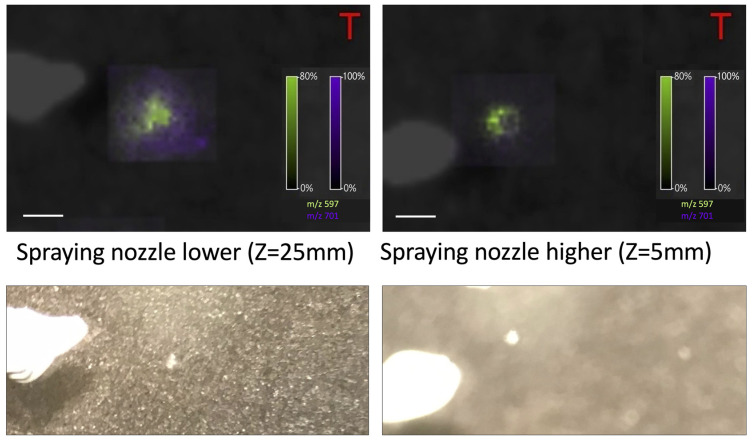
The picture of the oocyte obtained after matrix application at a lower nozzle position (Z = 25 mm) and higher nozzle position (Z = 5 mm), for the negative ionization mode, size bar: 200 μm. Below it is presented the microscopic image of oocytes.

Considering the spraying nozzle height for the negative mode, it seems that the lowest and the highest nozzle position allow to observe the peaks of high intensity (see: Figure 9 and Figure 10).

### 3.2. Different Matrix and Solvent Concentration

**Figure 11:**
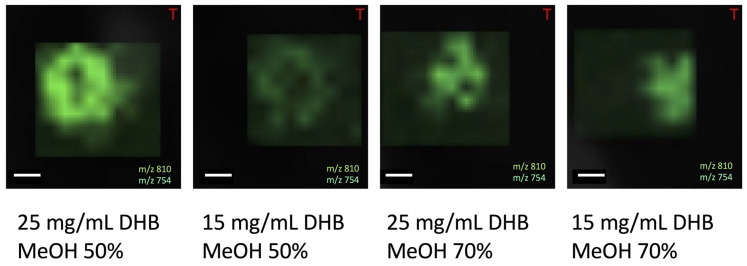
The picture of the oocytes obtained after covering them with matrix of different concentrations and solvent compositions, size bar: 100 μm.

During the next step, we tried to assess the influence of matrix concentration and the kind of matrix solvent for the analysis results. In the positive ionization mode, we have tested 25 mg/mL DHB in 50% and 70% methanol, and 15 mg/mL DHB in the same solutions. In this experiment classical composition (25 mg DHB, MeOH 50%) worked the best (see Figure 11).

We have also tested acetonitrile as a solvent, since it is sometimes used with DHB matrix, but in this case it does not work well. There were no reasonable peaks from lipids on the mass spectrum. For some of the *m*/*z* values the picture could be created, but it had very low quality.

**Figure 12:**
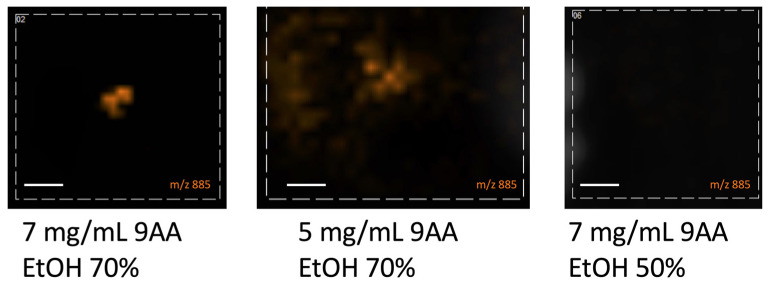
The picture of the oocyte obtained after covering them with the matrix of different concentrations and solvent compositions—negative ionization mode, size bar: 200 μm.

For the negative ionization mode, we used two 9AA matrix concentrations (5 mg/mL and 7 mg/mL), and two ethanol concentrations (50% and 70%, both *v*/*v*). The oocytes were covered with 14 layers of matrix, and the spraying nozzle height was established at Z = 1 mm. Here the classical choice of 7 mg/mL 9AA, 70% EtOH was also the best choice (see: Figure 12).

Methanol solution does not work for this kind of analysis with a 9AA matrix. It is impossible to observe lipids when such a solution is used.

### 3.3. Sample Preparation: PBS and PFA Fixing

For the measurements, oocytes were prepared in two ways. In the first approach, they were washed from the PBS (storage buffer) by immersing them for a few seconds in three subsequent drops of ultrapure water before deposition on ITO glass. In the second approach, oocytes were fixed by immersing them for 15 min in 4% PFA before washing in ultrapure water (3 times as well).

**Figure 13:**
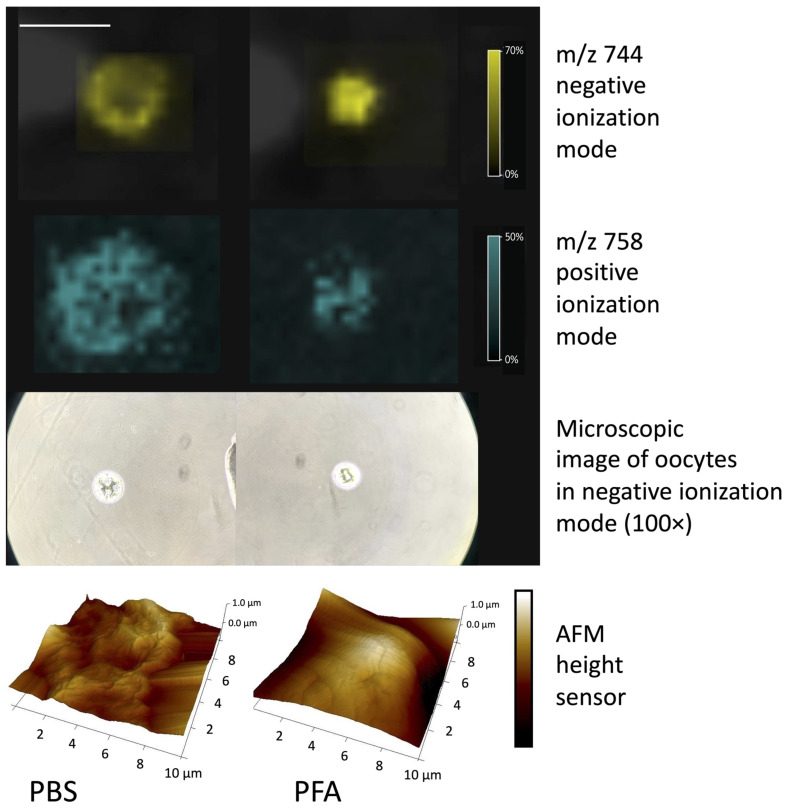
Oocyte washed with PBS (**left**) and the oocyte fixed with 4% PFA (**right**), negative and positive ion mode, with microscopic images of oocytes in the negative ionization mode. Additionally, AFM (Atomic Force Microscope) measurement of both types of the sample, to check of the morphology differences between cells, also visible by the MALDI-IMS. Size bar for images: 500 μm.

MS-imaging reveals the difference between these two ways of sample preparation. In Figure 13 (see also Supplementary Materials for step-by-step preparation sequence of the Figure 13), the oocyte washed with PBS is flattened on the ITO glass, and its content seems to be released, whereas the oocyte fixed with PFA retains its shape. The conclusions drawn from the MSI analysis were confirmed by AFM (Atomic Force Microscope) measurements. AFM analysis of oocyte washed with PBS showed severely damaged cell morphology that has lost its spherical shapes (Figure 13 left). On the other hand, for PFA, we observed unaltered oocytes with a spherical shape and smooth surface.

For PFA-fixed oocytes, it should be easier to mark the region of measurement and, thus, the measurements should be more reliable. On the other hand, the content of the cell on the glass seems to produce a higher, more reliable signal and it is easier to obtain the spectrum, especially in the negative ionization mode. To prevent the content of the cell from splashing, the highest (Z = 1) nozzle height should be used while measuring the oocytes just washed in water from PBS. On the other hand, to be able to obtain the signal from fixed oocytes with PFA, the lowest (Z = 25) nozzle height should be used.

## 4. Discussion

Measuring and imaging a single cell, even as huge as a single oocyte, is an analytical challenge, especially using a technique with many variables that may influence the result. In our approach, we decided that the overall high peak intensity on the spectra and good quality of obtained picture of the lipid distribution will be the main indicator of the quality of the proposed settings for the analysis. Additionally, we have tried to reveal some general rules influencing the quality of the MALDI-MSI measurements. During our analysis, we tested two matrices that are used in positive and negative ionization modes for the lipid measurements.

In both matrices, four different positions of the spraying nozzle above the sample surface and four different numbers of the matrix layers on the quality of obtained MS spectra were tested. In the positive ionization mode, the intensity of the peaks increases with the height of the nozzle. We also observed that lower nozzle height could be responsible for the unwanted “splashing” of the cell. Thus, the highest nozzle height (Z = 1) and ten (l = 10) matrix layers were chosen for the positive ionization mode.

For the negative ionization mode, the peak intensity increased with the number of the matrix layers as for the positive ionization mode. Thus, fourteen layers (l = 14) were chosen to be optimal. In the negative ionization mode higher nozzle position (Z = 1) could be used when oocytes are only stored in PBS and washed with water. However, to obtain a signal from PFA-fixed oocytes, lower nozzle height (Z = 25) seems to be necessary, especially in negative ionization mode.

We may speculate that the two phenomena influence the signal intensity when we consider the nozzle height over the sprayed surface. According to the thermal desorption mechanism proposed by Dreisenwerd [22,23], higher temperature increase desorption yield during the MALDI process. Smaller matrix crystals that are produced while spraying with the higher nozzle position achieve higher temperatures during the laser desorption process. It is due to the difficulty in the dissipation of laser energy connected simply with the smaller number of atoms in the crystal lattice and inefficient energy transfer between nearby crystals. However, proper extraction and cocrystallization are essential for some molecules and may be responsible for higher signal intensity. Here, the lower nozzle position is preferred, since it allows the production of wetter vapor that better penetrates the sample and allows for better cocrystallization. We may observe it, especially in the case of paraformaldehyde-fixed cells where paraformaldehyde produces cross-links between different chemical groups. It seems that the molecules need stronger extraction from the fixed cell, and that is why the lower nozzle position is able to produce the interpretable signal. Considering the number of matrix layers, it seems that the higher layer number is applied, the higher the signal intensity is obtained, to some point. Unfortunately, such optimization may be limited by the availability of precious material.

It is essential to mention that since in our samples we have plenty of molecules that may have different properties, the optimization procedures may indicate the trends characteristic for the particular sample source and may not be perfect for all samples.

Considering the matrix concentration and the organic solvent for the matrix in the positive ionization mode, we concluded that the classical matrix concentration and methanol as a solvent (25 mg/mL DHB, MeOH 50%) works the best. Changing the matrix solvent for ACN in the case of DHB does not allow for lipid measurement. The same is with the negative ionization mode, where the classic composition 7mg/mL 9AA and 70% EtOH works best.

**Figure 14:**
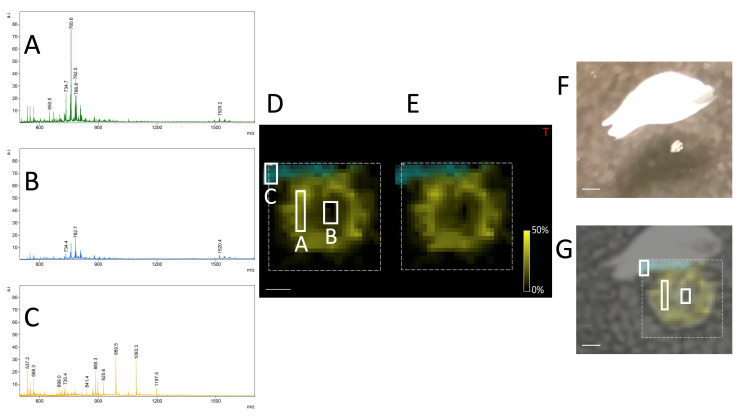
The comparison of the spectra obtained from different parts of the image of the oocyte washed with PBS (**A**)—spectrum from the splashed part of the cell (the intensity of the signal is increased), (**B**)—the signal directly from the cell (the inner part), (**C**)—the signal from the nail polish—the indicator of oocyte position, (**D**)—the picture of the oocyte with the marked region from which the spectra were created, (**E**)—overall view of the MSI image, (**F**)—the photo of the oocyte on the ITO glass, (**G**)—the photo of the same oocyte covered with matrix and overlapped by the MS image, size bar: 200 μm.

**Figure 15:**
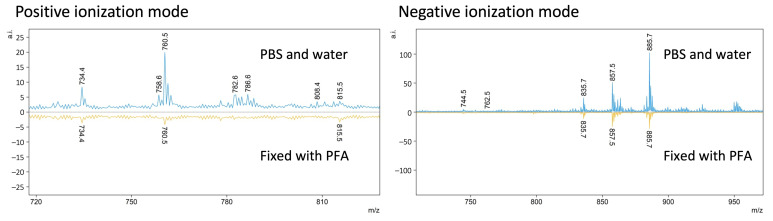
The comparison of the spectra of the oocytes washed with PBS and washed in PBA and fixed with PFA in the positive and negative ionization modes.

In the case of lipid analysis, fixing oocytes with PFA allows them to retain its shape. The content of the cell does not “splash” on the glass and makes the marking of the region of measurement easier and more reliable (see Figure 14 and also Supplementary Materials for co-registered images of the cell with a few ion maps for the different *m*/*z*). On the other hand, the signal from oocytes stored in PBS and washed with water is stronger and easier to obtain (see Figure 15). However, care must be taken to mark a bigger area around the cell. A higher nozzle height (Z = 1) should be used in this case to prevent the cell content from splashing over the glass. Thus, in this case, the measurement method may depend on the sample provided and the sensitivity of the mass spectrometer. Measuring the oocytes only washed with water might be advisable when we have an instrument of a lower sensitivity.

## 5. Conclusions

Due to the improvement of scientific techniques, especially because of their increased sensitivity, we can go deeper into the complicated biology of the single cell. This is important especially in the case of oocytes, where individual variability may be responsible for the fate of the oocyte and embryo development. The MALDI-MSI approach may analyze various kinds of lipids from a single oocyte. It is worth mentioning that this technique may be used to analyze embryos, which are more precious and harder to obtain as well.

To conclude, while using wet-interface matrix deposition techniques in the positive ionization mode with 25 mg/mL DHB, MeOH 50% matrix, the highest nozzle height (Z = 1) and ten (l = 10) matrix layers should be used. In the negative ionization mode, the 7 mg/mL 9AA 70% EtOH matrix works best. A high nozzle position (Z = 1) and fourteen matrix layers (l = 14) were also chosen to be optimal. Using oocytes washed from the PBS (storage buffer) by immersing them for a few seconds in three subsequent drops of ultrapure water before deposition on ITO glass allows us to obtain a higher, more reliable signal, especially in the negative ionization mode in comparison with the oocytes fixed with PFA.

We hope that our approach will be useful for lipid analysis in such extraordinary material.

## Data Availability

Data are available on request from the authors.

## Supplementary Materials

The following supporting information can be downloaded at: https://www.mdpi.com/article/10.3390/ma16041479/s1, Step-by-step preparation sequence of the Figure 13 from the manuscript and co-registered images of the cell with a few ion maps for the different *m*/*z*.
