# Supplementary material for: Mammalian Oocyte Analysis by MALDI MSI with Wet-Interface Matrix Deposition Technique
Source: Materials (Basel). 2023 Feb 9;16(4):1479. doi: 10.3390/ma16041479 (PMC9961235; doi:10.3390/ma16041479)
Supplement: Supplementary file 1 [file materials-16-01479-s001.zip › materials-2103924-supplementary.pdf]

Mammalian Oocyte Analysis by MALDI MSI with Wet-Interface Matrix Deposition Technique

Anna Bodzon-Kulakowska [1], Wiesława Młodawska [2], Przemyslaw Mielczarek [3], Dorota Lachowicz [4], Piotr Suder [1] and Marek Smoluch [1,*]

[1] Department of Analytical Chemistry and Biochemistry, Faculty of Materials Science and Ceramics, AGH University of Science and Technology, A. Mickiewicza 30, 30-059 Krakow, Poland
[2] Department of Animal Reproduction, Anatomy and Genomics, University of Agriculture in Krakow, Al. Mickiewicza 24/28, 30-059 Krakow, Poland
[3] Laboratory of Proteomics and Mass Spectrometry, Maj Institute of Pharmacology, Polish Academy of Sciences, Smetna 12, 31-343 Krakow, Poland
[4] Academic Centre for Materials and Nanotechnology, AGH University of Science and Technology, A. Mickiewicza 30, 30-059 Krakow, Poland

* Correspondence: smoluch@agh.edu.pl

Supplementary materials:

Step-by-step preparation sequence of the figure 13 from the manuscript

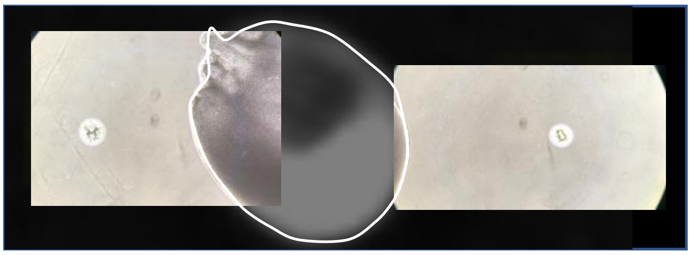

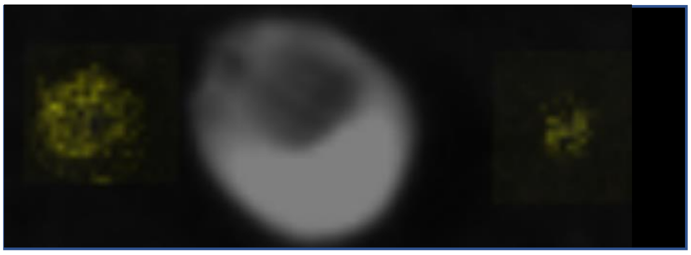

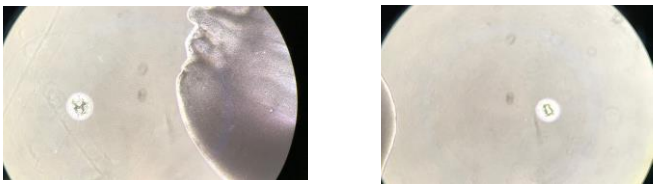

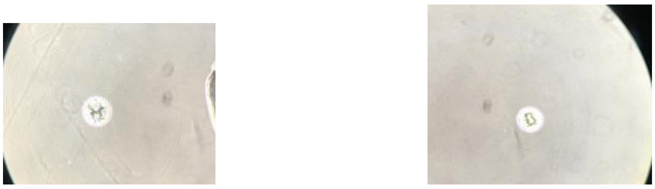

Original figure:

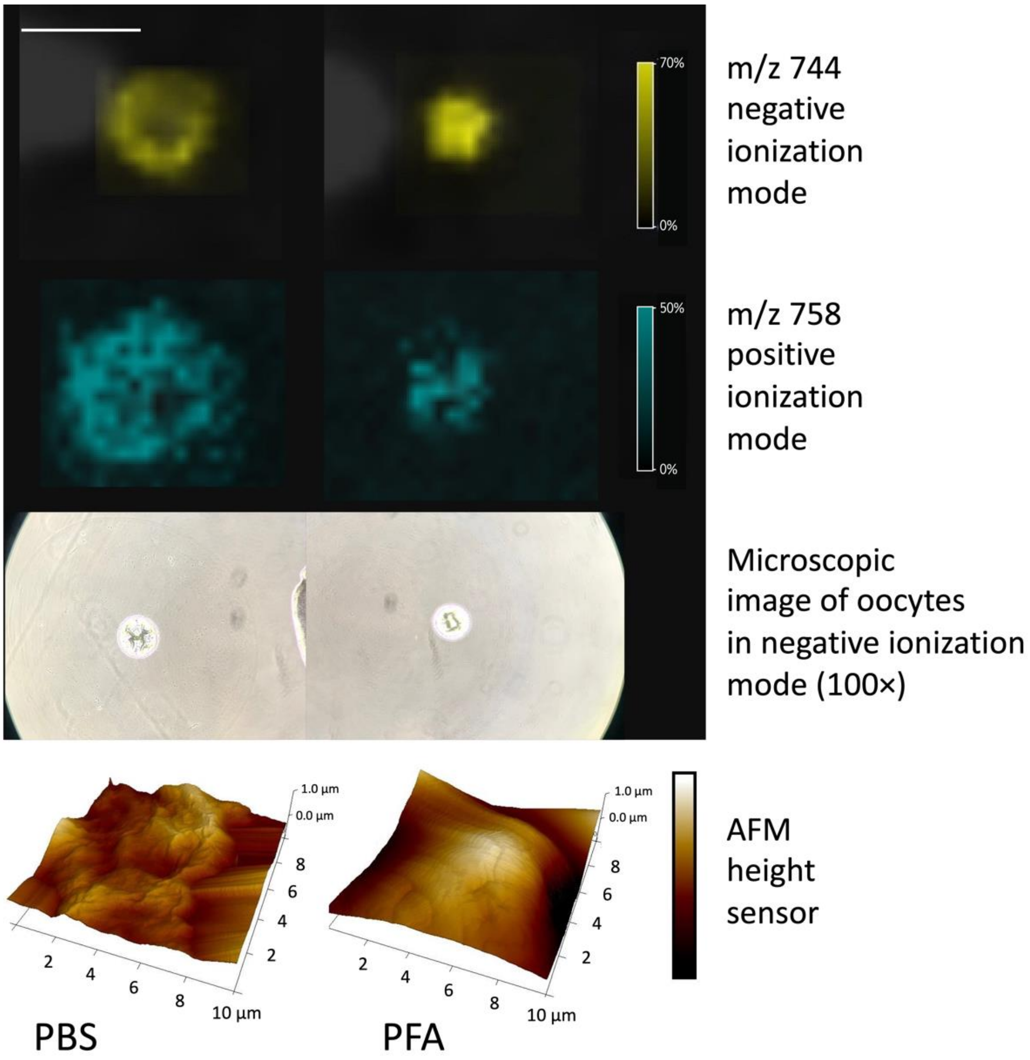

Figure S1. Oocyte washed with PBS (left) and the oocyte fixed with 4% PFA (right), negative and positive ionization mode, and AFM (Atomic Force Microscope) measurement of both types of the sample, size bar: 500 micrometers. Figure preparation.

Co-registered images of the cell with a few ion maps for the different m/z

Nozzle height: Z=15 mm, number of matrix layers: L=10

Before matrix deposition	Imaging	After matrix deposition and MS imaging

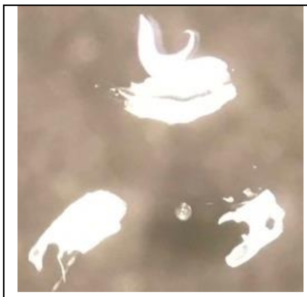	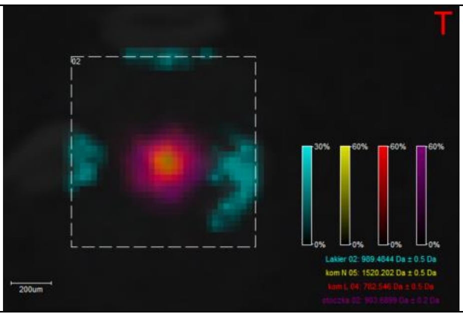	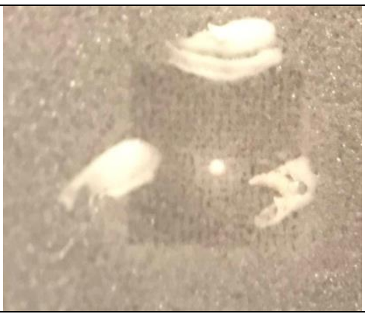

903.6 m/z	760.6 m/z	1520.2 m/z
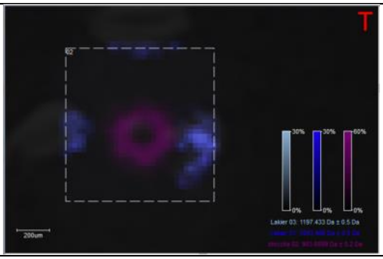	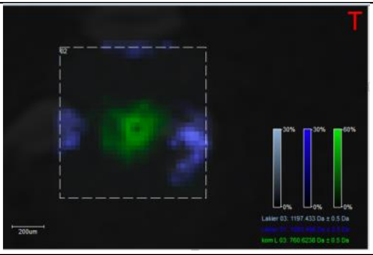	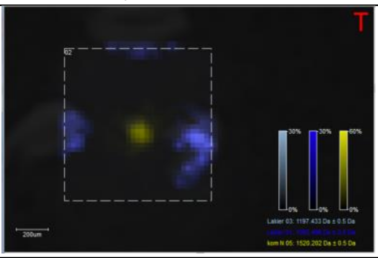
782.5 m/z	810.2 m/z	824.2 m/z
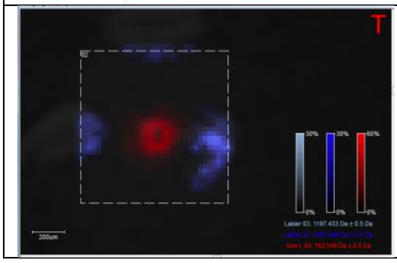	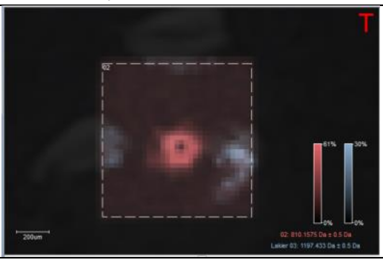	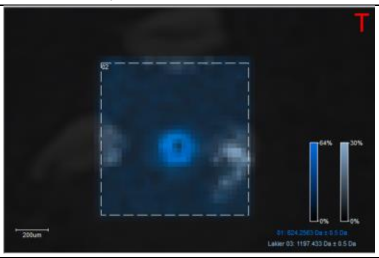

Nozzle height: Z=25 mm, number of matrix layers: L=10

Before matrix deposition	Imaging	After matrix deposition
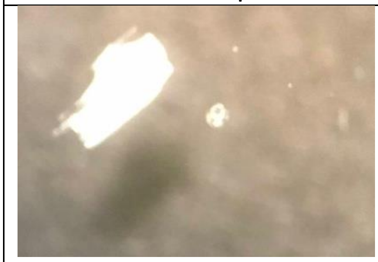	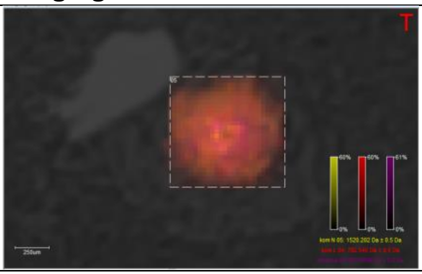	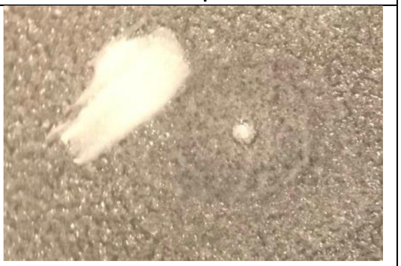

903.6 m/z	760.6 m/z	1520.2 m/z
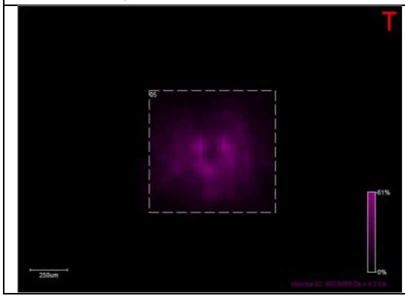	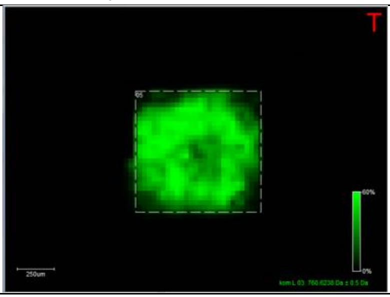	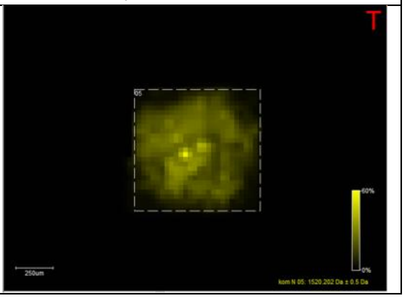

782.5 m/z	810.2 m/z	824.2 m/z
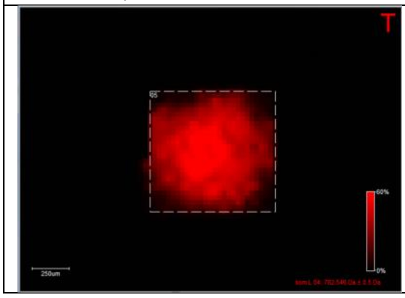	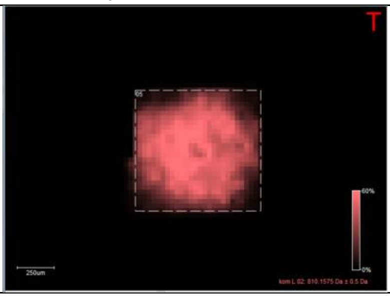	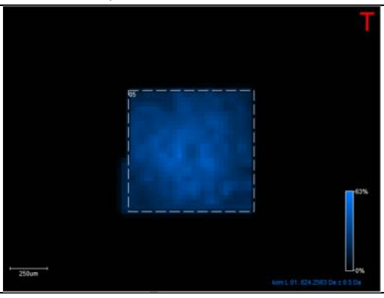
